# Supplementary figures and images for: Mir155 regulates osteogenesis and bone mass phenotype via targeting S1pr1 gene
Source: eLife. 2023 Jan 4;12:e77742. doi: 10.7554/eLife.77742 (PMC9839347; doi:10.7554/eLife.77742)

Figure 6C

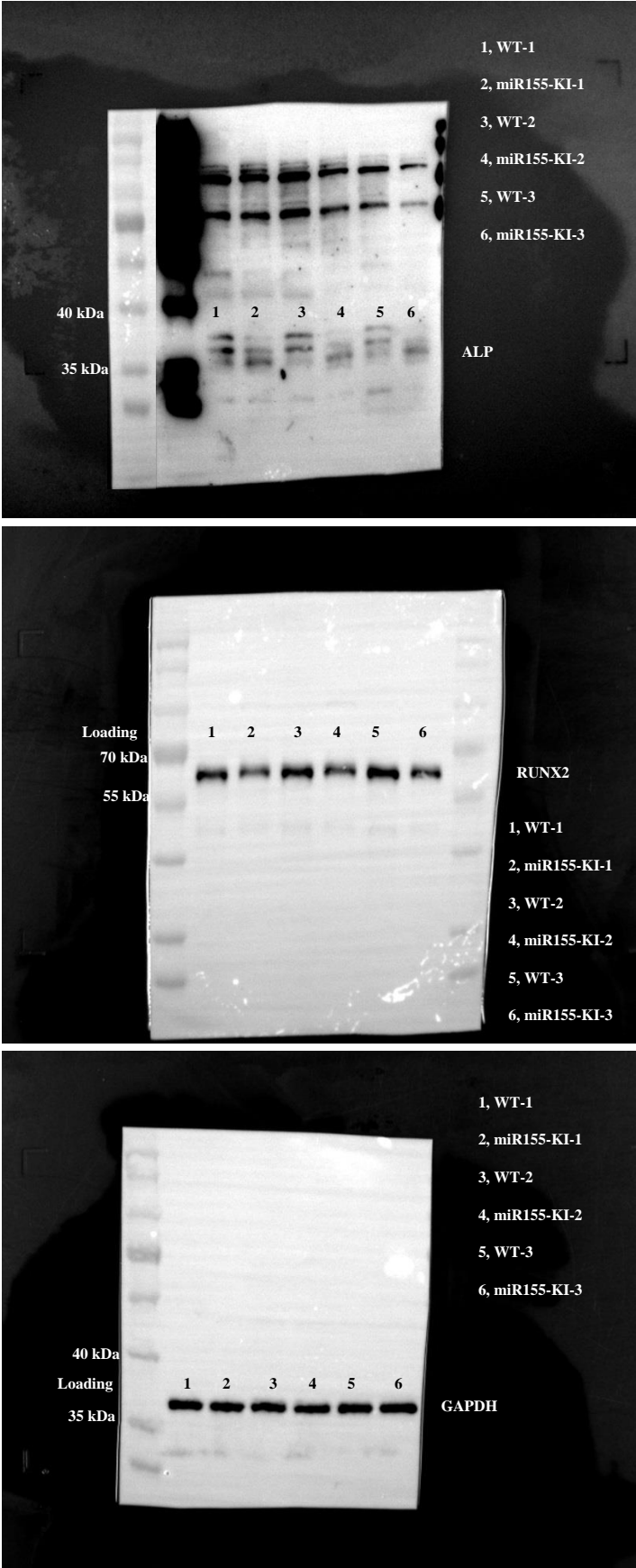

Figure 6G

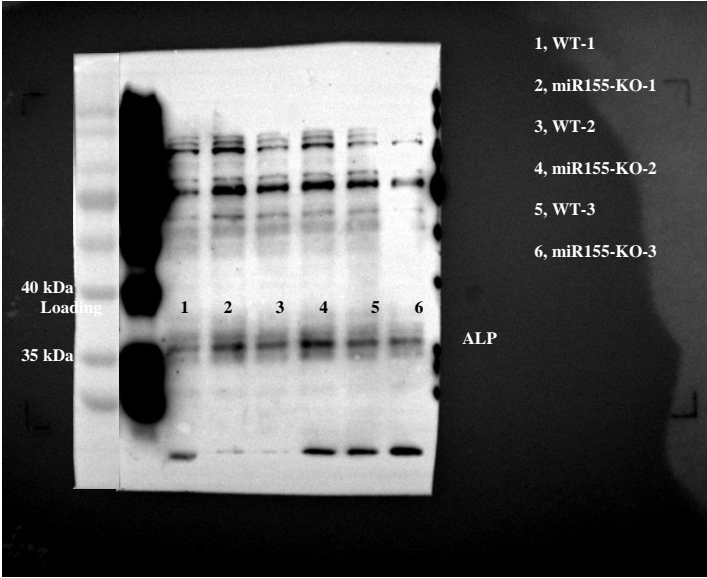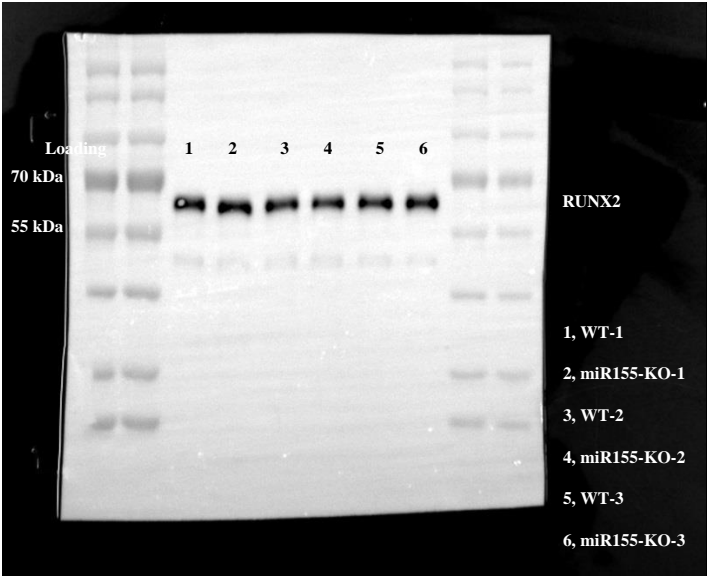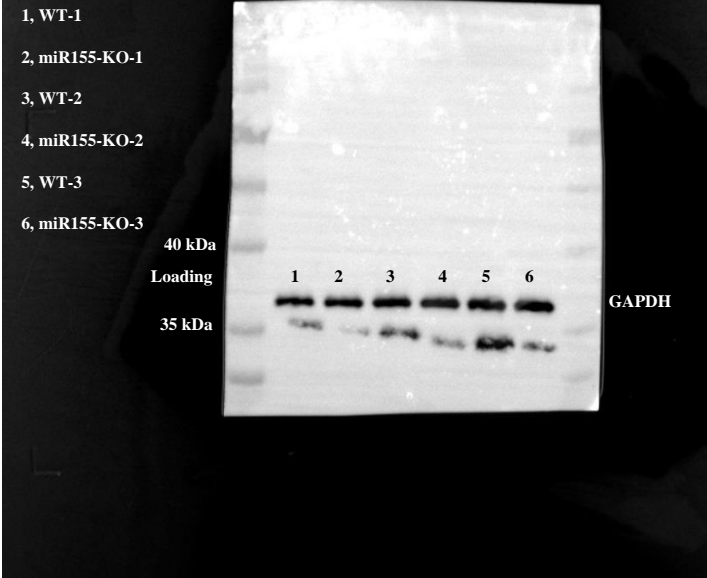

Supplement: Figure 6—source data 1. [file elife-77742-fig6-data1.zip › Original blots for Fig. 6C and 6G.pdf]

Figure 7D

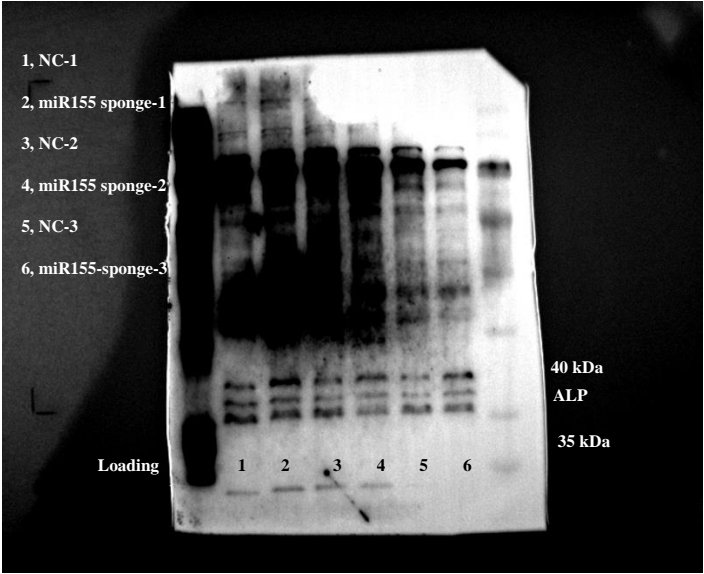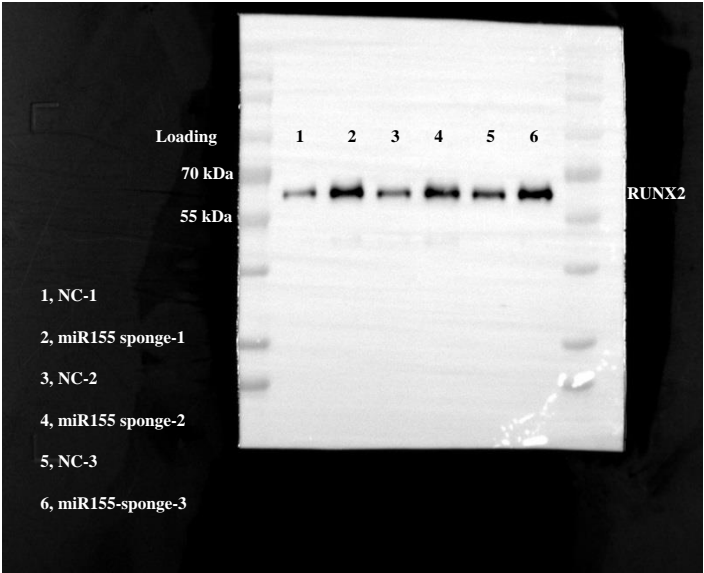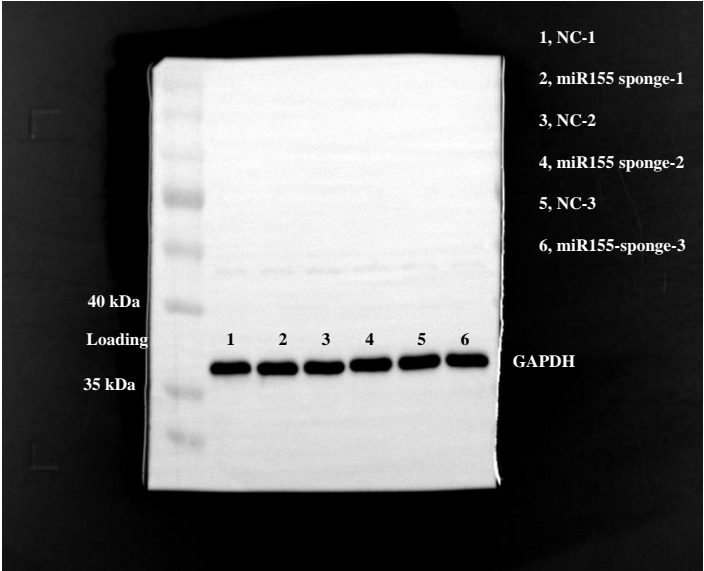

Supplement: Figure 7—source data 1. [file elife-77742-fig7-data1.zip › Original blots for Fig.7D.pdf]

Figure 8D

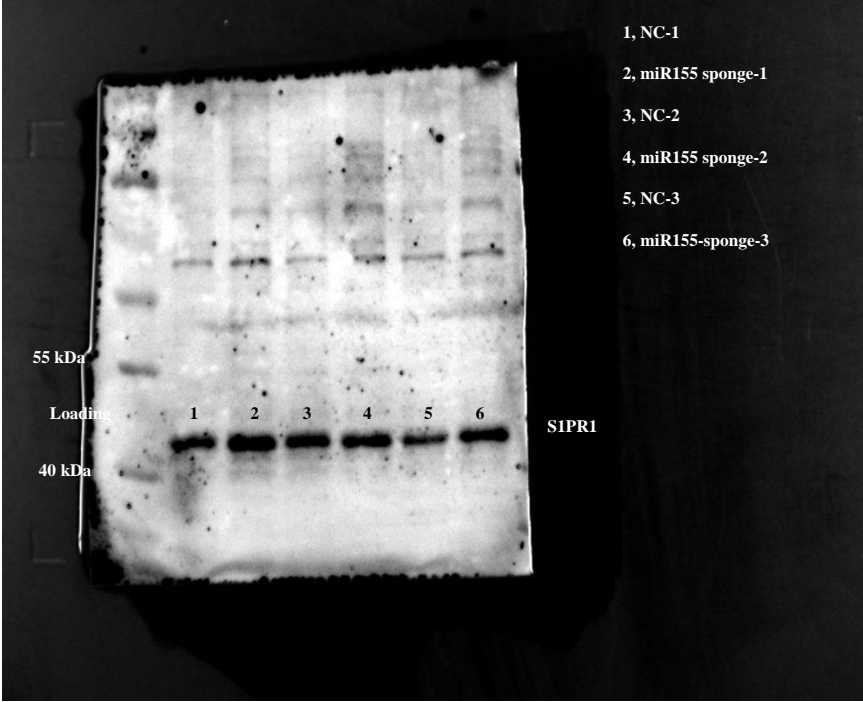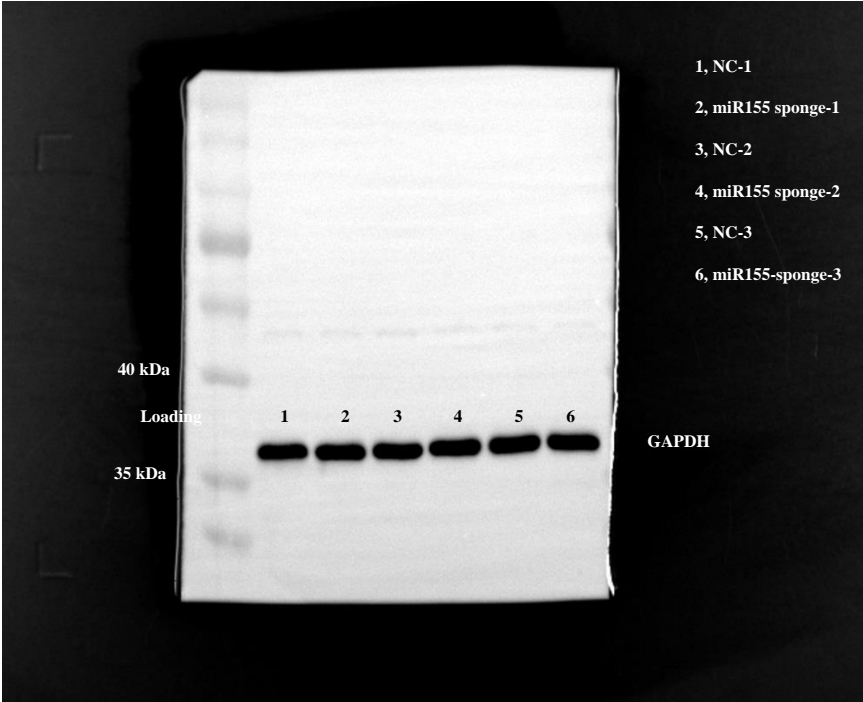

Figure 8G

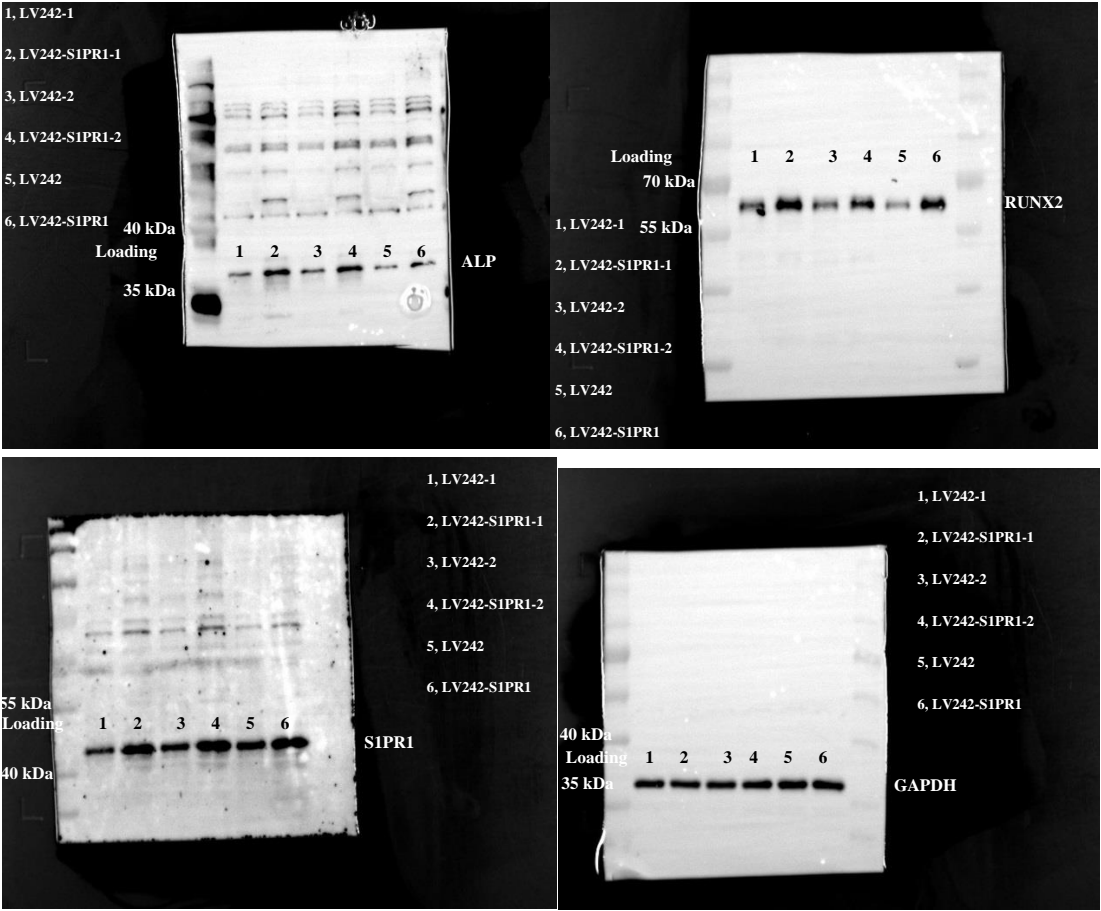

Supplement: Figure 8—source data 1. [file elife-77742-fig8-data1.zip › Original blots for Fig. 8D and Fig. 8G.pdf]
